# Burnout among Nursing Home Care Workers: The Influence of Work-Related and Personal Factors

**DOI:** 10.1093/geroni/igaf122.061

**Published:** 2025-12-31

**Authors:** Lisa Geyskens, Nasrin Khan, Anja Declercq, Koen Milisen, Mieke Deschodt

**Affiliations:** KU Leuven, Leuven, Vlaams-Brabant, Belgium; KU Leuven, Leuven, Vlaams-Brabant, Belgium; KU Leuven, Leuven, Vlaams-Brabant, Belgium; KU Leuven, Leuven, Vlaams-Brabant, Belgium; University of Leuven, KU Leuven, Leuven, Vlaams-Brabant, Belgium

## Abstract

Nursing homes face significant challenges due to increasingly complex care needs, and difficulties in recruiting and retaining care workers. High job demands and stressful work conditions contribute to burnout, exacerbating workforce shortages and potentially impacting care quality. This study describes the prevalence of burnout and its association with organizational, work environment, demographic, and intrapersonal factors among nursing home care workers. A secondary analysis was conducted using cross-sectional survey data from the Flanders Nursing Home (FLANH) project, including 1,521 care workers from 25 Flemish nursing homes. Participants included care assistants (43.7%), registered nurses (20.5%), support staff (15.4%), allied health professionals (14.8%), and team leaders (5.7%). Burnout was assessed using the short Burnout Assessment Tool (BAT-12), and associations were analyzed using a multivariable linear mixed model. Results showed that 19.0% of care workers were at risk of burnout. Higher staffing levels (β= -0.017, p = 0.0015), perceived staffing adequacy (β= -0.040, p = 0.0125), role clarity (β= -0.069, p = 0.0017), skill use (β= -0.109, p < 0.0001), and safety climate (β= -0.052, p = 0.0364) were significantly associated with lower burnout. Conversely, greater emotional burden at work (β = 0.085, p < 0.0001) and work-life interference (β = 0.266, p < 0.0001) were related to higher burnout. Older age, and higher self-efficacy, resilience, and optimism, emerged as significant protective factors. This study confirms the importance of adequate staffing and a supportive work environment in mitigating burnout among nursing home care workers. Targeted strategies to improve the work environment and strengthen intrapersonal resources are essential for promoting staff well-being and retention.

